# Supplementary material for: Predicting long-term functional anti-VEGF treatment outcomes in neovascular AMD in a real-world setting
Source: PLoS One. 2024 Nov 25;19(11):e0314167. doi: 10.1371/journal.pone.0314167 (PMC11588237; doi:10.1371/journal.pone.0314167)
Supplement: S1 Table — (DOCX) [file pone.0314167.s001.docx]

**S1 Table.** Effect sizes in univariate analyses for the variables in the final model

|  | **VA year 2** | | **VA year 3** | | **VA year 4** | | **VA year 5** | |
| --- | --- | --- | --- | --- | --- | --- | --- | --- |
|  | **ƒ^2^** |  | **ƒ^2^** |  | **ƒ^2^** |  | **ƒ^2^** |  |
| VA at baseline | **0.26** |  | **0.17** |  | **0.17** |  | **0.13** |  |
| CRT after loading | 0.01 |  | 0 |  | 0 |  | 0 |  |
| IRF at year 1 | 0.03 |  | 0.03 |  | 0.04 |  | 0.02 |  |
| Time to dryness | 0.02 |  | 0.04 |  | 0.02 |  | 0.02 |  |
| MA at year 1 | 0.19 |  | 0.21 |  | 0.18 |  | 0.21 |  |
| Interval extension | 0.03 |  | 0.04 |  | 0.01 |  | 0 |  |

According to Cohen’s (1988) guidelines, f^2^ ≥ 0.02, f^2^ ≥ 0.15, and f^2^ ≥ 0.35 represent small, medium, and large effect sizes, respectively (Cohen J. E. (1988). *Statistical Power Analysis for the Behavioral Sciences*. Hillsdale, NJ: Lawrence Erlbaum Associates, Inc). VA: Visual acuity in ETDRS letters with 1=85 letters.
