# Supplementary material for: Predicting long-term functional anti-VEGF treatment outcomes in neovascular AMD in a real-world setting
Source: PLoS One. 2024 Nov 25;19(11):e0314167. doi: 10.1371/journal.pone.0314167 (PMC11588237; doi:10.1371/journal.pone.0314167)
Supplement: S2 Table — (DOCX) [file pone.0314167.s002.docx]

**S2 Table**. Confidence intervals for the covariates included in the multiple regression model

|  | *Confidence Intervals* | |
| --- | --- | --- |
| **Dependent variable:**  **Visual acuity** | ***2.5%*** | **97.5%** |
| **After 2 years:** | |  |
| VA at baseline | 0.35 | 0.59 |
| CRT after loading | -0.02 | 0.01 |
| IRF at 1 year | -5.50 | 1.09 |
| Time to dryness | -0.19 | 0.12 |
| MA at 1 year | -17.32 | -9.56 |
| Interval extension | 2.92 | 8.86 |
| **After 3 years:** | |  |
| VA at baseline | 0.20 | 0.49 |
| CRT after loading | -0.02 | 0.01 |
| IRF at 1 year | -6.61 | 1.29 |
| Time to dryness | -0.30 | 0.04 |
| MA at 1 year | -21.55 | -11.72 |
| Interval extension | 2.99 | 10.09 |
| **After 4 years:** | |  |
| VA at baseline | 0.10 | 0.50 |
| CRT after loading | -0.03 | 0.01 |
| IRF at 1 year | -11.27 | -0.81 |
| Time to dryness | -0.24 | 0.19 |
| MA at 1 year | -24.52 | -11.03 |
| Interval extension | 1.04 | 10.79 |
| **After 5 years:** | |  |
| VA at baseline | 0.15 | 0.62 |
| CRT after loading | -0.02 | 0.02 |
| IRF at 1 year | -11.15 | 2.62 |
| Time to dryness | -0.30 | 0.22 |
| MA at 1 year | -29.73 | -12.75 |
| Interval extension | -0.40 | 11.60 |
