# Supplementary figures and images for: Predicting long-term functional anti-VEGF treatment outcomes in neovascular AMD in a real-world setting
Source: PLoS One. 2024 Nov 25;19(11):e0314167. doi: 10.1371/journal.pone.0314167 (PMC11588237; doi:10.1371/journal.pone.0314167)

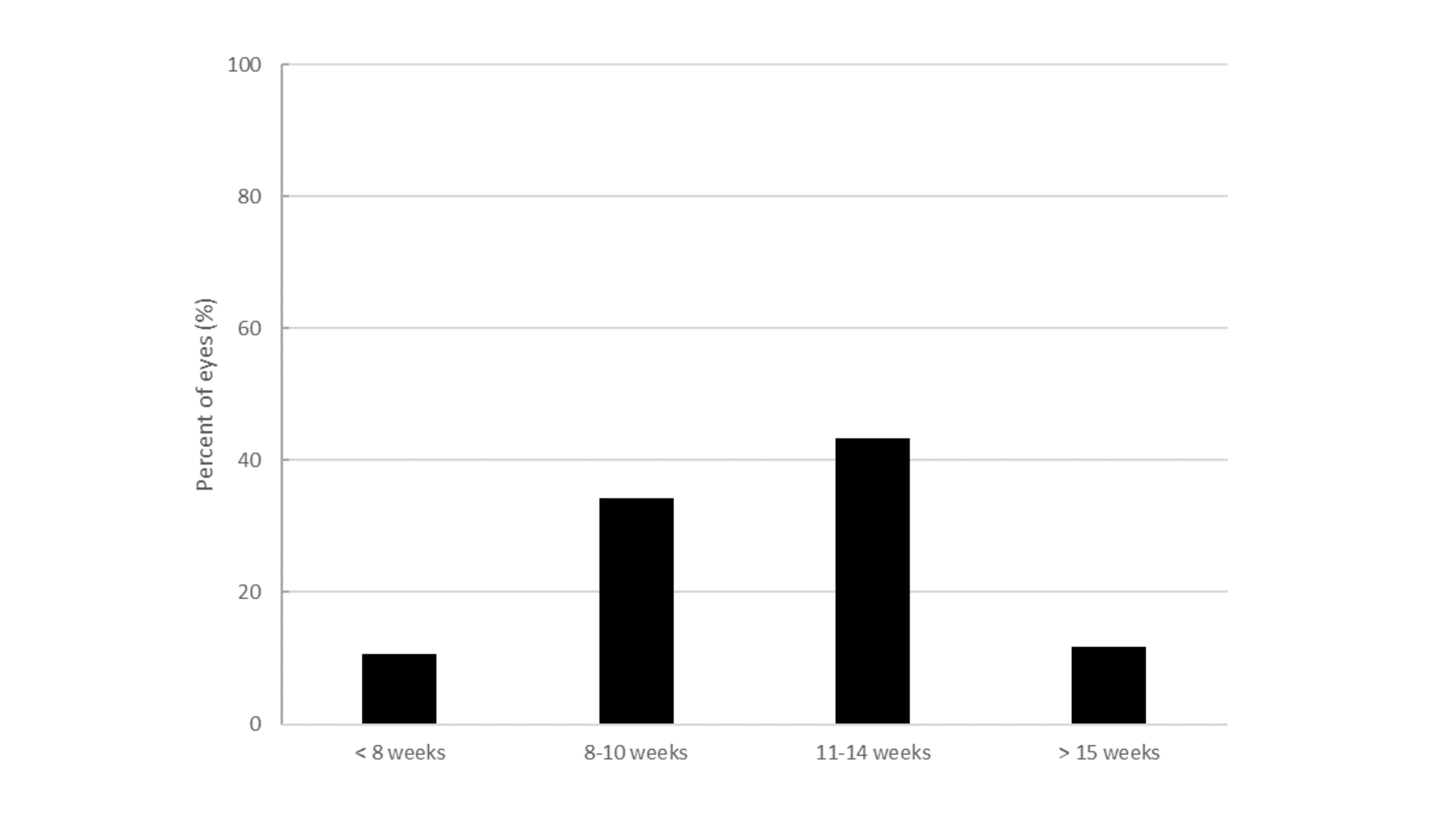

Supplement: S1 Fig — (TIF) [file pone.0314167.s003.tif]
